# Enacting pro-environmental behavior through grounded engagement: An interdisciplinary case study of plastic bag use in a Lao fresh market

**DOI:** 10.1016/j.isci.2026.115332

**Published:** 2026-03-12

**Authors:** Marco J. Haenssgen, Marieke Charlet, Serge Doussantousse, Kiyé Simon Luang

**Affiliations:** 1 Department of Social Sciences and Development, Chiang Mai University, Chiang Mai, Thailand; 2 Agence Universitaire de la Francophonie, Bureau national du Laos, National University of Laos, Dongdok Campus, Vientiane, Laos; 3 Green Vientiane, Vientiane, Laos; 4 KSL Studio, Vientiane, Laos

**Keywords:** Environmental science, Environmental issues, Social sciences

## Abstract

As we are reaching the limits of mono-disciplinary solutions for pro-environmental behavior change and single-use plastic reduction, this perspective documents how a recent development initiative formulated a bespoke behavior change portfolio in the Nonkho market of Vientiane Capital, Lao PDR. Our participatory approach was sensitive to gender, framed in a modern behavioral science framework, and drew on techniques from anthropology, development studies, economics, and the humanities. The resulting behavior change portfolio addressed people’s psychological capability, physical and social opportunity, reflective motivation, and various subconscious automatic drivers of plastic bag avoidance, all while promoting the social role and economic autonomy of female market vendors.

## Graphical abstract

**Figure:**
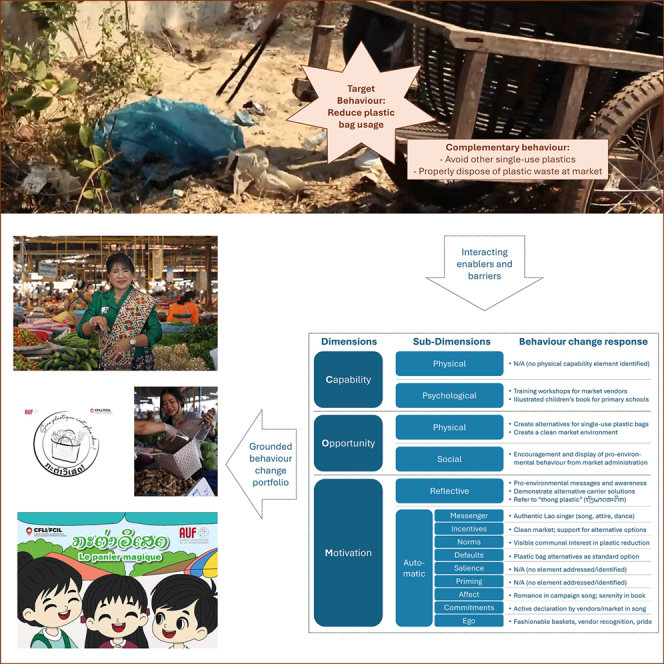

Aside from the potential to constitute intrinsically valuable outcomes of engaging, artistic and creative forms of expression can also facilitate understanding among, communication with, and behavior change of key audiences.

In line with the gender-focused aspect of the initiative – to realize development by women and for women – we recognized that markets in Lao PDR were heavily feminized spaces.

Vendors withholding plastic bags from shoppers would be considered “stingy” – a reputational impact that many did not wish to inflict on themselves.

The intentional use of varied basket designs in the video spoke to the automatic motivation element of “ego” as shoppers could present themselves fashionably during their visit.

Educational material for children in the book “*The Magic Basket”* fundamentally supported the “psychological capability” of pro-environmental behavior by generating an early didactic impulse to demonstrate and foster decision-making.

A comprehensive intervention package would complement [communication] efforts by addressing the physical opportunity of moving away from single-use plastics.

## Plastic pollution is a global and local challenge for sustainable development

Plastic pollution has advanced on environmental policy agendas as the world is bound to dispose a cumulative total of 12,000 mmt of plastic waste on landfills and in the natural environment by 2050.^1^ Alongside national and global action plans to combat plastic usage and pollution (e.g., national plastic bag bans, the Ocean Plastics Charter, or the European Strategy for Plastics in a Circular Economy), the policy significance of single-use plastics is visible in the commitment of all 193 United Nations member states to end plastic pollution and develop “an international legally binding instrument on plastic pollution.”^2^^,^^3^

Short-lived plastics, including single-use plastics, are a key component of this problem. Amounting to a global production volume of 238 mmt in 2019, single-use plastics include, for instance, food packaging or disposable carrier bags. These plastics have contributed to large-scale environmental pollution, global marine ecosystem disruption, and widespread microplastic contamination, with estimated social and environmental costs ranging from US$ 300 to 600 billion per year.^4^^,^^5^ Both the scale of unsustainable disposal (incl. e.g., open burning) and the social and environmental impacts of single-use plastic pollution are especially pronounced in low- and middle-income countries (LMICs).

This perspective contributes to a growing recognition that behavior change for environmental sustainability based on isolated communication efforts or decontextualized psychological interventions has exhausted its potential.^6^^,^^7^ Behavior change interventions in relation to single-use plastic require more comprehensive and grounded approaches that reflect the lived realities of affected populations and that account for the structural, cultural, and gendered dimensions of everyday plastic use.^8^^,^^9^ Recent advances in interdisciplinary behavioral science frameworks have opened new avenues for such context-sensitive intervention design,^7^^,^^10^^,^^11^ but few studies operationalize this logic in LMICs. Applied to the case of Lao PDR, this perspective illustrates how a grounded, participatory, and gender-sensitive approach can yield a bespoke behavior change portfolio to overcome the limitations of mono-disciplinary environmental communication campaigns, and thereby offers a model for more holistic, equitable, and culturally sensitive forms of pro-environmental engagement.

Lao PDR is a lower-middle-income landlocked developing country in Southeast Asia with a population of 7.6 million people.^12^ The Southeast Asian region where the Lao PDR is located is known for high levels of plastic waste mismanagement.^13^ And similar to development patterns across the region, the development progress of Lao PDR has accelerated the production of waste, which is expected to increase by more than 50% to 1.4 mt between 2020 and 2035.^14^ Detailed national statistics are unavailable and estimates vary, but much of the waste production appears to be driven by urban centers, where plastic waste accounts for around 16% of all collected waste.^14^ However, Lao PDR has low rates of waste collection in regional comparison (48% in Vientiane Capital in 2016 compared to 100% in Bangkok, 97% in Ho Chi Minh City, and 92% in Phnom Penh), and a large portion of solid waste is burned or dumped openly.^15^

Single-use plastics are a key driver of the Lao PDR’s environmental health challenges that result from this constellation, which has become visible in the microplastic contamination of marshlands, leakage of toxic chemicals into local ecosystems, and even increased flood risks from drains blocked by plastic waste.^14^ The Lao Ministry of Natural Resources and Environment^16^ recognises the severity of this issue in its *National plastic action plan for the Lao PDR 2024–2030*, which covers, among other activities, waste management regulations for single-use plastic bags and plastic bag bans in supermarkets from 23 May 2025 onwards.

However, the plastic crisis also has a distinct gender dimension. Development research in general has widely established that women tend to bear a high burden of unpaid work and maintain limited decision-making power in the household,^17^^,^^18^ which makes them more prone to be involved in plastic use as household consumers. Southeast Asia and the Lao PDR in particular reflect these conditions. Fresh markets in Lao PDR are well known to be gendered environments, and Lao society places patriarchal expectations on the economic activities and mobility of women.^19^^,^^20^^,^^21^^,^^22^ The situation in Lao PDR within the region is yet more pronounced as it has been assessed as one of the most gender-unequal countries in all of Asia-Pacific (27^th^ out of 37 countries).^8^

In recognition of the need to embrace the lived and gendered realities of plastic bag usage,^9^ the escalating plastic pollution problem, and its intersectional challenges, a pro-environmental development initiative was initiated with the objective to tackle single-use plastics in Lao PDR. The project focused specifically on single-use plastic bag usage as a particularly visible facet of the Lao PDR’s plastic challenge, which we explored in one of the largest fresh markets in Vientiane Capital City. The study team was led by Agence Universitaire de la Francophonie in Laos (AUF Laos), working in close collaboration with KSL Studio and the local environmental organizations Green Vientiane and Econox.

Written permission was obtained from the market owner, and informed consent for the voluntary participation in the media work and the foundational market engagement to define the intervention package was obtained from all participants.

## Environmental communication to tackle single-use plastic pollution requires interdisciplinary impulses

Addressing plastic pollution from single-use sources is a multi-faceted challenge. These facets involve, for instance, regulatory and technological responses such as sustainable production guidelines, the development of substitutes for plastic production, or plastic bag bans.^13^ It is also partly a behavioral challenge.^5^^,^^7^^,^^11^^,^^23^^,^^24^ As such, it requires, among others, “effective social and behavior change communication strategies to end plastic pollution.”^4^ Such communication activities have been shown to produce positive behavioral effects through personal communication such as emails,^25^ environmental announcements and posters,^26^^,^^27^^,^^28^ or environmental information using mass and social media.^29^^,^^30^^,^^31^^,^^32^^,^^33^

These environmental communication activities and associated research are often rooted in psychological frameworks, with social cognitive theory^34^ and the theory of planned behavior^35^ being among the most frequently applied behavioral frameworks in general and in environmental communication research specifically.^24^^,^^30^^,^^31^^,^^36^^,^^37^^,^^38^^,^^39^ These frameworks have found extensive empirical support and helped to depart from overly simplistic (and indeed patronizing) “knowledge deficit” approaches to behavior change, for instance by considering behavioral intentions as an intermediary stage between knowledge and action, or heterogeneously distributed beliefs in people’s ability to enact change.^6^^,^^40^^,^^41^^,^^42^

However, the limitations of the classical psychological framing of behavior are well-known and include implicit assumptions of rational decision-making, a methodological focus on the individual that is detached from broader social and political structures, and a claim to universality that undermines contextual grounding and culturally sensitive intervention design.^40^^,^^41^ It has been shown repeatedly that pro-environmental behavior is more complex and multi-layered: It competes with other livelihood priorities, impulses and habits, broader systems of oppression and privilege, and interacts with the broader context in which it is embedded. These issues become particularly visible in less privileged, non-western, and LMIC settings.^6^^,^^43^^,^^44^^,^^45^

Modern behavioral sciences approaches have synthesized lessons and theoretical arguments from diverse disciplines beyond psychology, such as neuroscience, economics, sociology, and anthropology. The currently most influential framework is the Capability-Opportunity-Motivation-Behavior (COM-B) model.^10^ Its advantages include the recognition that reflective rationality as embraced by classical psychological models accounts for approximately one-fifth of people’s decision-making processes, while the remainder of our decisions are driven by automatic cognitive processes that include heuristics, habits, and impulses.^41^^,^^46^^,^^47^ The MINDSPACE framework (an acronym comprising common mechanisms of automatic motivation: messenger, incentives, norms, defaults, salience, priming, affect, commitment, and ego) summarizes nine common mechanisms that drive these automatic process.^47^

However, all of this is provided that a decision can be made at all. For example, if pro-environmental behavior involves putting “used papers into recycle bins,”^31^ then this does not only depend on environmental information (reflective motivation), habitual behavior and stress (automatic motivation), and broader social norms around recycling (social opportunity), but such bins also need to be available (physical opportunity) and accessible (physical capability). Although not intrinsic to the model, the open-endedness and context sensitivity of COM-B also allow analysts to explore broader political and historical drivers of behavior, for instance, oppressive gender norms or problematic histories with the state that can undermine the effectiveness of top-down pro-environmental information provision.^9^

This is not to say that previous environmental communication research has not addressed such broader aspects of behavior change in (implicit) accordance with the various COM-B categories. Recent studies have explored personal interests and values shaping behavioral outcomes,^48^ message framing,^25^^,^^29^ the influence of different messengers,^49^ affective elements such as feelings of guilt or nostalgia,^26^^,^^50^ harms (i.e., loss aversion),^32^ different modes of communication such as immersive virtual reality techniques,^51^ social norms and the socio-cultural context of decision-making,^27^^,^^52^ or the broader information environment to which individuals are exposed through mass and social media.^30^^,^^31^^,^^32^ Psychologically informed research is also not intrinsically insensitive to potential unintended consequences of information campaigns,^7^^,^^53^ which have been explored, for instance, in environmental communication studies in the US context.^28^^,^^54^^,^^55^

However, these explorations have proceeded largely in a piecemeal fashion and without comprehensive and grounded assessments of existing environmental practices and their underlying rationale. A key advantage of broader behaviorally informed research that conducts such assessments is that they do not *a priori* prioritize communication over other options in the behavior change toolkit (e.g., reconfiguration of the physical environment to make options available, subconscious behavioral nudges through scents or subtle appeals to pride).^7^^,^^10^^,^^56^ Rather, intervention design is founded on the grounded analysis of behavioral systems and barriers and enablers therein, on the basis of which a portfolio of targeted responses to support the collaboratively identified target behaviors can be formulated.^7^^,^^11^

Doing so requires an engaged approach to behavior change interventions. To some extent, all communication activity directed at the general population could be framed as “public engagement.”^57^ However, interventions that are designed from the outset with communication as the solution tend to be less “engaged” and more instrumental than those that identify in consultation with local stakeholders and populations whether communication tools are promising instruments within the broader behavioral system.^58^

Interdisciplinary approaches involving the arts and humanities can support this venture. Aside from the intrinsic value of artistic products that the humanities typically foreground, creative and artistic products are often part of broader science communication at the dissemination stage of research projects (often under the headings of “public engagement” or “science communication”).^59^ Arts-based approaches can also be directly integrated in communication and intervention strategies, thus supporting behavior change among key audiences through place-based and contextualized engagement with environmental issues.^60^^,^^61^ Although this approach is less common due to the open-ended and unpredictable nature of creative forms of expression,^60^^,^^62^ it can create measurable outcomes with potential for sustained behavioral change.^57^^,^^60^^,^^63^ The present case study in Lao PDR speaks to this latter approach.

In the specific context of Southeast Asia, interventions to reduce plastic waste have mostly revolved around policy packages comprising technological, systemic, and top-down solutions together with institutional capacity building, for example integrated waste management systems and waste bank programmes in Indonesia, the expansion of biodegradable materials in Thailand, or supply-side interventions such as an environmental protection tax since 2010 and extended bans of single-use plastics in Vietnam from 2026 onwards.^13^^,^^64^^,^^65^ As the effectiveness and public acceptability of these schemes so far has been rather mixed, this case study from Lao PDR offers a complementary perspective on behavioral aspects of plastic usage from the bottom-up.

## A participatory approach in an urban Lao market

In order to attain this grounded understanding of plastic-related practices with sensitivity to gender and with the conscious inclusion of creative forms of expression, our project team needed to go beyond the conventional behavior-change-communication toolkit. The interdisciplinary approach in our development initiative involved the active recognition of women both as informants and development agents for positive change. As a participatory development initiative, the work focused on remedying a local environmental challenge rather than “research,” as a result of which it did not correspond to requirements for research ethics approval. However, all work was carried out with formal permission from the market owner, and informed consent for the voluntary participation in the media work and the foundational market engagement to define the intervention package was obtained from all participants.

Methodologically, the approach drew on insights from the social sciences (development studies, social anthropology, economics) and the humanities to inform and respond to behavioral challenges that are conventionally addressed from a psychological and/or communication studies perspective. The combination of foundational methods to develop the behavior change package involved:

• 5 consultative discussions and a training workshop with 51 vendors on how to reconcile profitability and environmental responsibility in their business (led by Econox and Green Vientiane),
• Participant observation of market practices,
• 40 video interviews with market vendors, customers, and the market owner, and
• A survey of a random sample of 203 market vendors to understand the relationship between plastic bag usage and vendor characteristics in a nine-item digital questionnaire (collected in a random walk with ten random starting points within the market).

We combined participatory, qualitative, and quantitative methods under an exploratory approach, meaning that the survey data served to contextualize and expand rather than replace grounded qualitative insights. The project was implemented between November 2024 and March 2025 by the AUF-Laos-led project team, supported by videographer Kiyé Simon Luang (KSL Studio), ten students from the National University of Laos and the University of Health Sciences, and in collaboration with the environmental organizations Green Vientiane and Econox. The student team also received specialized training in survey data collection and the digital survey tool in the “Le Sphinx” app (www.lesphinx.es). All engagement took place in the Lao language by native speakers.

The qualitative materials were analyzed in an inductive thematic fashion to contextualize the study environment, document plastic use practices and their influences, and to identify actionable elements for the development of a pro-environmental behavior change basket.^66^ The quantitative data were analyzed descriptively to discern broader patterns of plastic bag use and their covariates within the market setting,^67^ complemented by an exploratory model of predicted plastic bag usage depending on the characteristics of market sellers (see statistical note in the Supplemental Material).^68^

**Figure 1:**
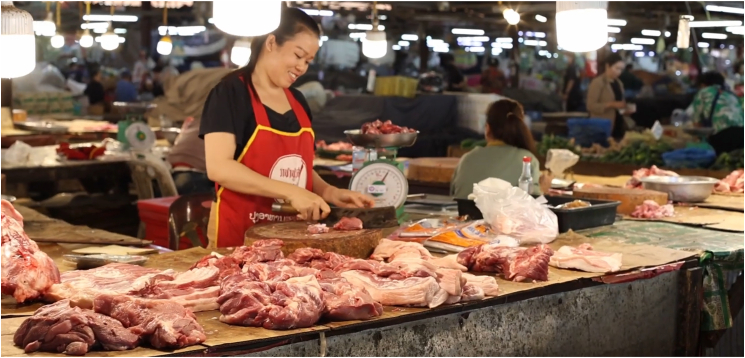
Impression of the Nonkho market in peri-urban Vientiane Capital of Lao PDR

The work took place in Nonkho market (Figure 1), which was one of the largest fresh markets in Lao PDR, located in a peri-urban area of Vientiane. The market had a surface area of 2.5 ha, over a thousand stalls, and over 3000 customers daily. It operated as a wholesale market at night and as a retail market during the day. In line with the gender-focused aspect of the initiative – to realize development by women and for women – we recognized that markets in Lao PDR were heavily feminized spaces. Specifically, Laotian women were largely responsible for household purchases, food preparation, and managing household finances. Given the strong link between women’s domestic activities and the fresh market, women residing in nearby villages constituted 80% of the market vendors and the majority of its customers, while men primarily handled transportation, wholesale deliveries, and market maintenance. The market owner was a female entrepreneur as well.

Prior to selecting the Nonkho market, the project team gauged the potential demand for an environmentally oriented development project. The market owner actively supported the project, facilitated all planned activities, and echoed the project’s potential to enhance the market’s reputation and customer traffic by recognizing and empowering women. Vendors also expressed their willingness to participate in interviews and training. However, the participatory approach also required the project team to understand and accommodate the daily demands faced by the market vendors (i.e., to “come their way”) to enable meaningful involvement in the discussions and formulation of responses to local plastic use challenges.

## Groundwork to identify the challenges of plastic bag usage in Nonkho Market

Preliminary engagement to develop the behavior change campaign demonstrated that single-use plastic bags were a large-scale and deeply entrenched challenge. The survey established a baseline of practices: The participating 203 vendors used, on average, 144.65 plastic bags per day, with a median usage of 100 bags/day (range from 10 to 900; see Supplemental Material for details). At these rates, between 6.5 and 9.4 *million* plastic bags were used at the market per year (assuming 360 work days and 600 vendors per year on average), costing vendors US$ 300 to 430 per year (LAK 3.89bn to 5.64bn; exchange rate of LAK/US$ = 21680.86 by www.xe.com as of 3 March 2025). These expenses correspond roughly to a one-month salary of a trained medical doctor in Lao PDR. A substantial amount.

Average daily plastic bag usage across different types of stalls ranged from 104 bags/day for clothing stalls to approximately 160 bags/day for stalls selling dried food, fresh vegetables, or “other” products. Plastic bags were the most conspicuous single-use plastic product on the market, but other common types of plastics at the market also included plastic cups, straws, and foam packaging for food. Several stalls at the market sold these items exclusively. Littering of plastic and other waste was pervasive, without suitable waste storage and management facilities at the market.

The participating vendors were interested in reducing plastic bags, but our engagement identified several factors that drove and reinforced the large scale of plastic bag usage. One key aspect was a common lack of knowledge surrounding the extent of plastic pollution. This was partly owed to language issues: The typical Lao term for “plastic bag” is “thong yang” (ຖົງຢາງ), which implies that the bags are a natural product made from rubber or latex (“yang” or ຢາງ). Only 29% of the 51 vendors who participated in the workshop were aware that plastic bags were not sourced from rubber trees but derived from oil. Accordingly, most vendors would not consider environmental impacts when using plastic or otherwise assume that discarded plastic would be collected by others. Only a few would acknowledge problems of plastic waste and environmental concerns relating to toxic contaminants, air pollution, and waste accumulating in streets and rivers.

In practice, despite some explicit interest in reducing plastic bag usage, vendors remained unenthusiastic. A key reason was the difficult-to-replace convenience of plastic bags and other plastic products: Consider, for instance, the ease of hanging a plastic bag for a beverage in a take-away cup from a motorcycle handle. Another concern was vendors’ fear of revenue loss. Observations by our project team indicated that plastic bags were often seen as a sign of financial success, signifying the successful completion of a transaction. Conversely, vendors withholding plastic bags from shoppers would be considered “stingy.” Many vendors did not wish to inflict such a reputational impact on themselves.

Moving away from the plastic bag standard would require overcoming these established practices, and the need for viable alternatives, without which vendors would likely incur at least some financial loss. Seemingly easy alternatives, such as cloth or paper bags, were not commonly available while also proving more costly than plastic bags. This would render financial support essential to adopt alternative solutions, alongside more general guidance and support that vendors requested explicitly. Alternative carrier options were also at times seen as inferior: Vendors explained that, “[filled] *with meat*, a *paper bag tears,*” and that, “*Our products are heavy and require plastic bags.*” Without these practical and affordable alternatives, the majority of 71% of the 203 surveyed market vendors would not deem it possible to reduce plastic bag use in Lao fresh markets.

## Developing a pro-environmental behavior change basket to reduce plastic bag usage

In response to the observed patterns and drivers of single-use plastic bag usage, the study team and market vendors together sought options to strengthen the economic autonomy of market vendors while promoting environmental responsibility through reduced plastic waste.

**Figure 2:**
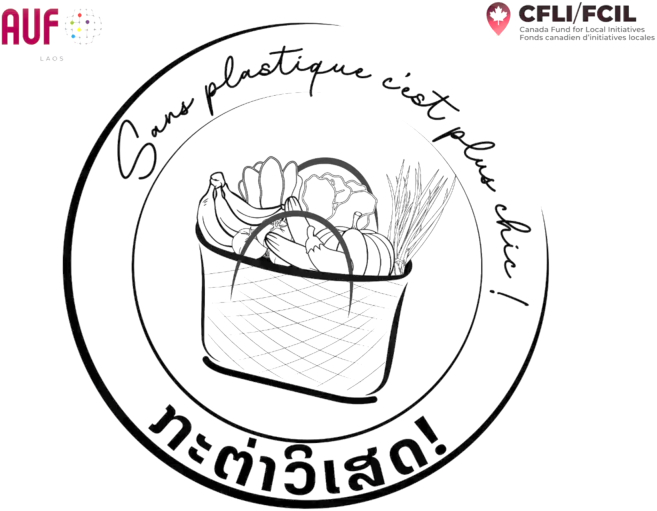
The logo representing the pro-environmental behavior change communication campaign “*No plastic, it’s more chic!*”

The initial response to changing plastic-related behavior was the development of a pro-environmental behavior change communication campaign. Carrying the slogan “*No plastic, it’s more chic!*” (Figure 2), the campaign contained several creative approaches (materials are available from authors), including:

• The exclusive creation of the song “*The Young Woman from Nonkho Market*” (www.youtube.com/watch?v=XR1gjdZ9oqc) (singer: Mee Sayphousam; lyrics: Yartfa Kavisin; composition and recording: Daovong Detkumman; production: Marieke Charlet, AUF Laos; director: Kiyé Simon Luang, KSL Studio; actors: market vendors and students from the National University of Laos and University of Health Sciences),
• A 15-min documentary film produced by ten students from the National University of Laos and the University of Health Sciences in collaboration with filmmaker Kiyé Simon Luang (KSL Studio; https://www.youtube.com/watch?v=wunq0ev4LtQ),
• Training sessions for vendors conducted by the local environmental organizations Green Vientiane and Econox,
• 5 shows of traditional singers and dancers inside the market with environmental messages, and
• The production of an illustrated children’s book called “*The Magic Basket,*” with 4,500 copies set to be distributed in primary schools across Lao PDR to educate young students on the environmental and health impacts of plastic waste.

The song, including its video showcasing Nonkho market, was one of the most visible elements in the project’s communication portfolio. It addressed several key aspects of reflective and automatic motivation among both vendors and shoppers directly (see lyrics and video stills below). Reflective decision-making aspects were addressed through explicit **pro-environmental messages** (“*Bring your baskets! Help us preserve the environment*.”) and the **demonstration of basket use as an alternative solution** to plastic bags. These elements were designed to inform conscious decisions, such as the weighing of different carrier options when shoppers visit the market.

**Figure 3:**
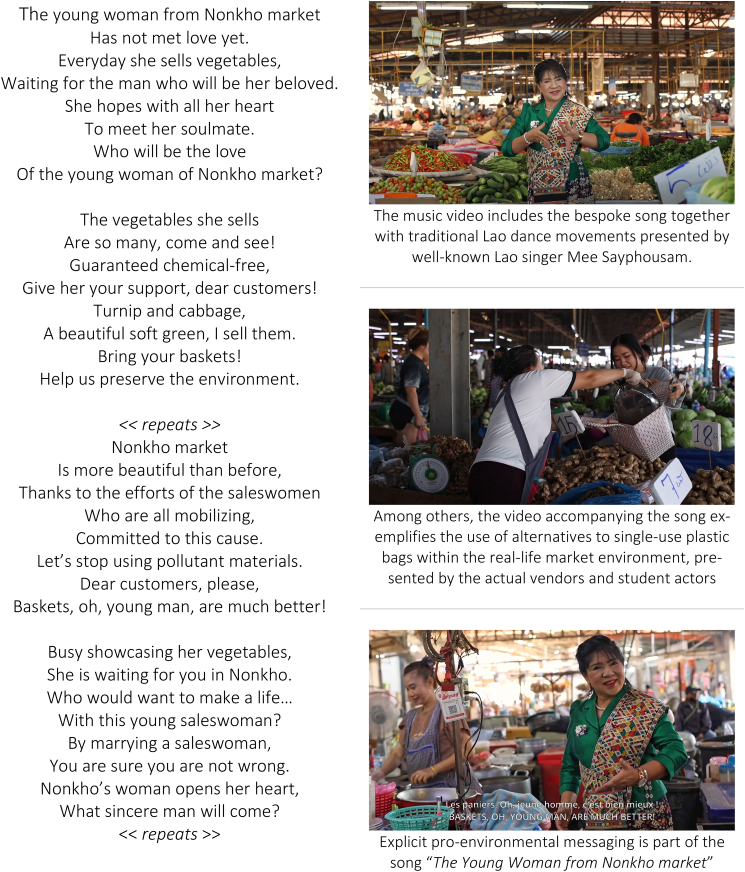
Lyrics and video stills from the song “*The Young Woman from Nonkho market*,” which was developed specifically for the single-use plastic bag study at Nonkho market *Notes.* Singer: Mee Sayphousam; lyrics: Yartfa Kavisin; composition and recording: Daovong Detkumman; production: Marieke Charlet, AUF Laos; director: Kiyé Simon Luang; actors: market vendors and students from the National University of Laos and University of Health Sciences.

Because well-meaning pro-environmental messages are only weakly effective when used in isolation, the song also addressed at least five automatic motivation elements to reduce single-use plastic bags (Figure 3).^47^ The most visible strategy was the involvement of well-known female Lao singer Mee Sayphousam (www.youtube.com/@meesayphousam4918). Her appearance in traditional Lao attire and her performance of traditional dance moves reinforced her role as a relatable and authentic “**messenger**” to convey the environmental messages to the primarily female market audience – as opposed to messages relayed by external environmental experts (which are often conveyed through male voices) or Western, Thai, or East Asian performers. The romantic theme of the song appealed to the behavioral element of “**affect**” in motivating our choices positively, while the video also alluded to a broader acceptance of pro-environmental behavior through “**norms**” as it depicted the uncontroversial use of baskets and indicated that market actors “*are all mobilizing*” for a reduction of plastic bag usage. In addition, the intentional use of varied basket designs in the video spoke to the automatic motivation element of “**ego**” as shoppers could present themselves fashionably during their visit. “**Ego**” was also reinforced on the side of market vendors as the video has been designed to nurture their pride as female merchants in Nonkho market. Lastly, the song activated the “**commitment**” element of automatic motivation as it publicly reported the Nonkho saleswomen as “*committed to this cause,*” thus rendering their pro-environmental behavior open to the scrutiny of shoppers and their peers.

**Figure 4:**
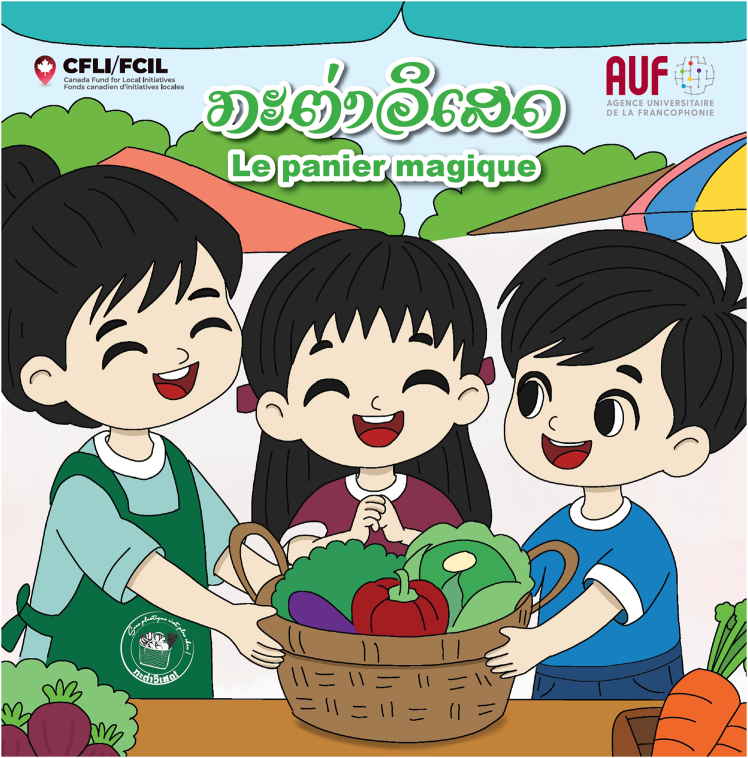
Cover image of the educational children’s book “The Magic Basket.” The book tells the story of Pok and Ta, who solve the domestic plastic bag problem by starting to use a basket when buying groceries at the local market.

Other elements of the campaign also spoke to behavioral drivers in the COM-B model. For example, the educational content for children in the book “*The Magic Basket*” (Figure 4) fundamentally supported the “**psychological capability**” of pro-environmental behavior by generating an early didactic impulse to demonstrate and foster decision-making. The book tells the story of the children Pok and Ta, who observe plastic bag pollution and its health impacts on their domestic animals, environment, and family. In response, the children decide to start using a basket instead of the ubiquitous plastic bags. While demonstrating the children’s ability to assess and respond to a local environmental challenge (and thus building psychological capability), the narrative alludes to the visible environmental and health costs of plastic bags that motivated the decision (**reflective motivation**). It also addresses several elements of automatic motivation as it is intentionally relatable to children’s living environments (**“norms”** and **“messenger”**), it underscores the “*pride*” with which the children carry their basket (**“ego”**), and it concludes with feelings of serenity as “*mom welcomes them with a smile*” under a tree shining full of “*ripe yellow mangoes*” upon their return from the market (**“affect”**). In addition, the documentary and related social media activity addressed “**reflective motivation**” in a similarly explicit way as the music video content. Even the slogan and logo of the campaign itself are intended to raise awareness (**reflective motivation**) while activating shoppers’ “**ego**” (**automatic motivation**) through the “chic” self-image of someone who does not use plastic bags. In addition, the project team insisted on using the term “thong plastic” (ຖົງພາດສະຕິກ) to refer to plastic bags during their activities so as to raise awareness about the origin of these products.

The grounded and participatory approach involving female vendors also enabled several other behavioral considerations beyond the communication campaign. First, the grounded approach recognized that many vendors worked long hours in noisy environments, making engagement challenging. Shoppers and vendors were also highly diverse. For example, small dried food stalls, large clothing and drinks stalls, and both small and large stalls selling fresh fruit/vegetables and meat/fish gave away large numbers of plastic bags (see Supplemental Material), but only a minority of the vendors (esp. in clothing and drinks stalls) deemed it possible to reduce plastic bag usage. Especially stalls selling raw meat and fish would face immediate hygiene and consumer acceptability challenges when moving away from customary plastic bags. Only one-sixth of these, therefore, considered plastic bag reductions realistic. Market shoppers agreed that “*otherwise the basket is dirty*” if they tried not to use plastic bags. This meant that stalls selling clothes and drinks/cooked food might be most receptive to initiatives to reduce plastic bags, but they only represented 19% of the market stalls. Fruit and vegetable stalls accounted for nearly 40% of the vendors at the market, but their involvement might require more extensive demonstration efforts and the availability of alternative carrier options. Observations at the market suggested that vendors who exclusively sold single-use plastic products were key engagement partners with special (and particularly challenging) economic circumstances for single-use plastic reduction.

In addition, “**norms**” for plastic avoidance among vendors and shoppers (representing the social environment while their perception is also part of automatic motivation) could be boosted by the project and market staff leading by example; that is, conspicuously avoiding the use of single-use plastic bags, cups, and bottles at the market. Likewise, a vendor recognition program could select and reward the most environmentally conscious vendors each month, hence reinforcing community “**norms**,” boosting vendors' self-image (“**ego**”), and providing positive “**incentives**” for pro-environmental practices (all drivers of automatic motivation). The market administration could also play a role in recruiting dedicated staff focused on cleanup and plastic reduction efforts, while developing waste storage and composting facilities, as well as re-use and recycling options for plastic waste. In combination, these activities would help better manage plastic waste on the market (i.e., contributing to a supplementary target behavior) and improve overall market cleanliness (thereby influencing the **physical environment** and increasing the relative loss avoidance costs or dis-“**incentives**” of littering in the market with single-use plastics).

**Figure 5:**
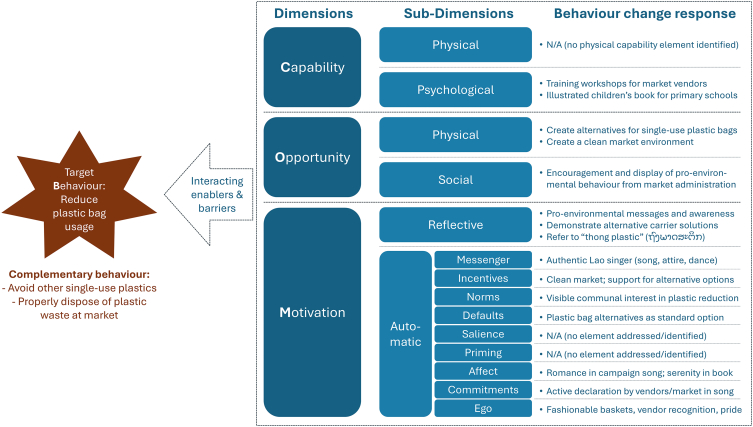
Summary of the portfolio of behavior change responses to single-use plastic bag usage at Nonkho market within the COM-B model domains

A comprehensive intervention package would complement these efforts by addressing the **physical opportunity** of moving away from single-use plastics. Such options include compostable plastic bags (and bio-degradable alternatives for other single-use plastic products) as well as cloth bags, paper bags, and baskets – not merely as part of the communication campaigns but also making them physically available at the market (e.g., for sale, as communally shared objects, lent by vendors). However, vendors would require economic/financial support for some of these alternative options where their sales or operating costs would be affected (e.g., more expensive paper bags or compostable plastic bags). This would help mitigate barriers in the **physical environment** and reduce loss aversion (“**incentives**”). At the same time, vendors providing plastic bag alternatives would also leverage the habitual “**default**” behavior of shoppers, as many do not actually have an insatiable desire to carry vast amounts of plastic bags but rather “go with the flow” of the provided convenient option. Figure 5 summarizes these considerations within the COM-B model.

## Conclusions

We are reaching the limits of the seemingly “easy” mono-disciplinary solutions for pro-environmental behavior change and single-use plastic reduction. Interdisciplinary and participatory approaches can help us recognize the complexity of behavior and the limitations of single-strand interventions,^7^^,^^9^^,^^56^ while the arts and humanities can inspire creative forms of expression to facilitate the engagement with key audiences.^60^^,^^61^ A portfolio of behavior change levers based on these approaches can address plastic-related practices from the bottom-up, resulting in a basket of activities that is not limited to explicit awareness messages but also addresses cognitive processes as well as personal, social, and other contextual factors in people’s decision-making environment. Our case demonstrated how such a portfolio can emerge from participatory engagement in a fresh market with a high concentration of single-use plastics, and it can potentially serve as a process model for intervention designers in other contexts.

The design opportunities utilized in this initiative demonstrate that we can overcome the plateauing gains in conventional pro-environmental communication. While prior work has shown how message framing, messenger identity, and emotional appeals can influence pro-environmental behaviors,^25^^,^^26^^,^^29^^,^^49^^,^^50^ much of this research has assumed that communication is the primary or only lever of change. Intersectional, structural, and contextual challenges have particular weight in LMIC settings where communication alone cannot address infrastructural gaps, financial constraints, or cultural norms.^42^ The interdisciplinary and grounded approach in our development initiative helped highlight such competing challenges. Our study team was exposed to the simultaneous livelihood struggles of the saleswomen (for whom and by whom the responses were developed) and the important role of single-use plastics for many practical problems at the market (e.g., hygienic handling and transport of fresh meat). The gender component of this work further balanced the behavior change portfolio as it emphasized the parallel objective of promoting women’s economic autonomy: by recognizing their socio-economic role in Lao society and by showcasing their capacity as market vendors to create income-generating activities. Our work thereby demonstrates how straightforward environmental communication is merely one of several tools within a behaviorally grounded portfolio.

Practitioners who consider adopting our approach should note that some elements of this case study are highly context specific, including:

• the format and content of the activities: for example, the specific language, media, imagery, and metaphors employed in the communication and educational materials,
• the physical and social nature of the fresh market environments and surrounding infrastructure: for example, visitors arriving on a motorcycle and buying unpacked meat and fresh vegetables in conditions with variable hygiene levels, and
• gendered experiences of plastic usage: high gender inequality in Lao PDR with persistence of patriarchal norms alongside a majority of women as market vendors and customers.

Other low-, middle-, and high-income countries may present different configurations of the existing intervention landscape, policy momentum, socio-economic constraints, cultural practices, or gender empowerment. However, the design principles incorporated in this case study are transferable as they help respond to the specific social, cultural, economic, technological, and political contexts of plastic bag use. Emulating this process would involve:

1. Foundational participatory assessment of behavioral systems, ideally supported by mixed-methods baseline studies and in recognition of gendered realities of plastic use (alongside other dimensions of intersectionality).
2. Holistic identification of behavioral drivers and enablers of plastic bag (or other single-use plastics) usage; beyond knowledge deficits and in the recognition of automatic decision-making, physical and psychological opportunity, and physical and social factors in the broader behavioral setting.
3. Co-design of interventions with local stakeholders, including members of the target audiences, to ensure cultural and economic appropriateness, and without unduly placing the burden of change on economically strained or otherwise disadvantaged target populations. Co-design should directly and systematically address the relevant elements in the behavioral system and consider opportunities for arts-based interventions.
4. Formulation of broader intervention packages that integrate harmoniously into the plastic waste/circular economy policy landscape and the broader structural barriers and enablers of pro-environmental behavior, such as livelihood challenges and gender discrimination.

The way forward in the Lao PDR requires attention to three challenges. First, sustaining pro-environmental behavior beyond the initial enthusiasm sparked by a campaign requires mechanisms for long-term reinforcement and adaptation. Second, scaling bottom-up participatory interventions to the regional or national levels depends on institutional support and flexibility to accommodate diverse local contexts. Third, gender empowerment and market-centric approaches must be complemented with efforts to address systemic and infrastructural barriers as well as with behavioral change targeting up- and down-stream actors (e.g., policymakers, professional plastic collection and compost advocates, consumers) so that environmental responsibility does not unfairly pressure women and market vendors.

To sustain the momentum of the initiative, we therefore encourage Nonkho market to adopt a formal environmental strategy that institutionalizes the behavioral change elements of this initial activity and the “*No plastic, it’s more chic!*” campaign to form new low-plastic habits and showcase positive environmental norms. Nonkho market may establish itself in the medium term as Lao PDRs’ first “low-plastic market” while acknowledging that the complete elimination of plastics from markets is not feasible. Efforts to expand the participatory behavior change approach to other sites in Lao PDR will greatly benefit from coordination with the Lao Ministry of Natural Resources and Environment and the *National plastic action plan for the Lao PDR 2024–2030* to ensure coherence not only with behavioral and social but also policy systems. Beyond market vendors, the lateral expansion of the initiative will further require the involvement of market owners to take a leadership role and foster a sense of ownership in the growing movement toward plastic waste reduction. In addition, so as not to place the burden unduly on female market vendors, a need for broader community and district participation will be crucial to sustain and reinforce the behavior change portfolio systemically and over the long term.

## Acknowledgments

We wish to thank the Canada Fund for Local Initiatives for financial support and trust, AUF Asia-Pacific, and the Embassy of Canada in Laos for institutional support, Nonkho Market administration for logistical facilities, financial support, and market access, and we acknowledge with deep gratitude the fruitful interactions with our project collaborators KSL Studio, Green Vientiane, Econox, and Mee Sayphousam. We further thank the vendors and market visitors for inspiring exchanges that contributed to the development of the arguments and ideas encapsulated in this manuscript.

Funding: This research was funded by the Canada Fund for Local Initiatives (contract ref. CFLI-2024-BNGKK-LA-0013). The funders had no role in project design; in the collection, analysis, and interpretation of data; in the writing of the report; and in the decision to submit the article for publication.

## Declaration of interests

The authors declare no conflict of interest.
