# Supplementary material for: Enacting pro-environmental behavior through grounded engagement: An interdisciplinary case study of plastic bag use in a Lao fresh market
Source: iScience. 2026 Mar 12;29(5):115332. doi: 10.1016/j.isci.2026.115332 (PMC13145880; doi:10.1016/j.isci.2026.115332)
Supplement: Document S1. Supplemental methods [file mmc1.pdf]

iScience, Volume *29*

Supplemental information

Enacting pro-environmental behavior through grounded engagement: An interdisciplinary case study of plastic bag use in a Lao fresh market

Marco J. Haenssgen, Marieke Charlet, Serge Doussantousse, and Kiyé Simon Luang

Supplemental Material

Summary in French

Alors que nous atteignons les limites des solutions monodisciplinaires pour encourager des comportements pro-environnementaux et réduire l'utilisation de plastique à usage unique, cette rétrospective retrace comment une initiative de développement récente a conçu une approche de changement social et comportemental sur mesure dans le marché de Nonkho, à Vientiane, capitale du Laos. Cette action participative, sensible aux questions de genre, s'inscrivait dans un cadre moderne de sciences comportementales et s'appuyait sur des techniques issues de l'anthropologie, des études du développement, de l'économie et des sciences humaines. L'approche de changement social et comportemental ainsi élaborée ciblait la capacité psychologique des individus, les opportunités physiques et sociales, la motivation réflexive ainsi que divers mécanismes automatiques et inconscients influençant l'évitement des sacs plastiques – tout en valorisant le rôle social et l'autonomie économique des vendeuses du marché.

Summary of market survey

Variable		*n*	Mean	Std. dev.	Min	Max
Plastic bags per day		203	144.65	135.06	10	900
Spending on plastic bags per month (LAK)		203	359,901.50	372,322.40	20,000	2,500,000
Vendor thinks it possible to reduce plastic bag use in Lao fresh markets (1 = yes)		203	28.57%	0.45	0	1
Main products sold	Clothing	203	7.39%	0.26	0	1
	Dried or processed food	203	14.29%	0.35	0	1
	Drinks and cooked food	203	11.33%	0.32	0	1
	Fresh meat or fish	203	21.18%	0.41	0	1
	Fresh vegetables and fruits	203	38.42%	0.49	0	1
	Other	203	7.39%	0.26	0	1
Stall size	Half square	203	43.35%	0.50	0	1
	Full square	203	46.80%	0.50	0	1
	Other size	203	9.85%	0.30	0	1
Age of vendor		203	41.57	11.98	14	70
Gender of vendor	Female	203	79.80%	0.40	0	1
	Male	203	18.72%	0.39	0	1
	Other	203	1.48%	0.12	0	1

Daily plastic bag usage by vendor attributes

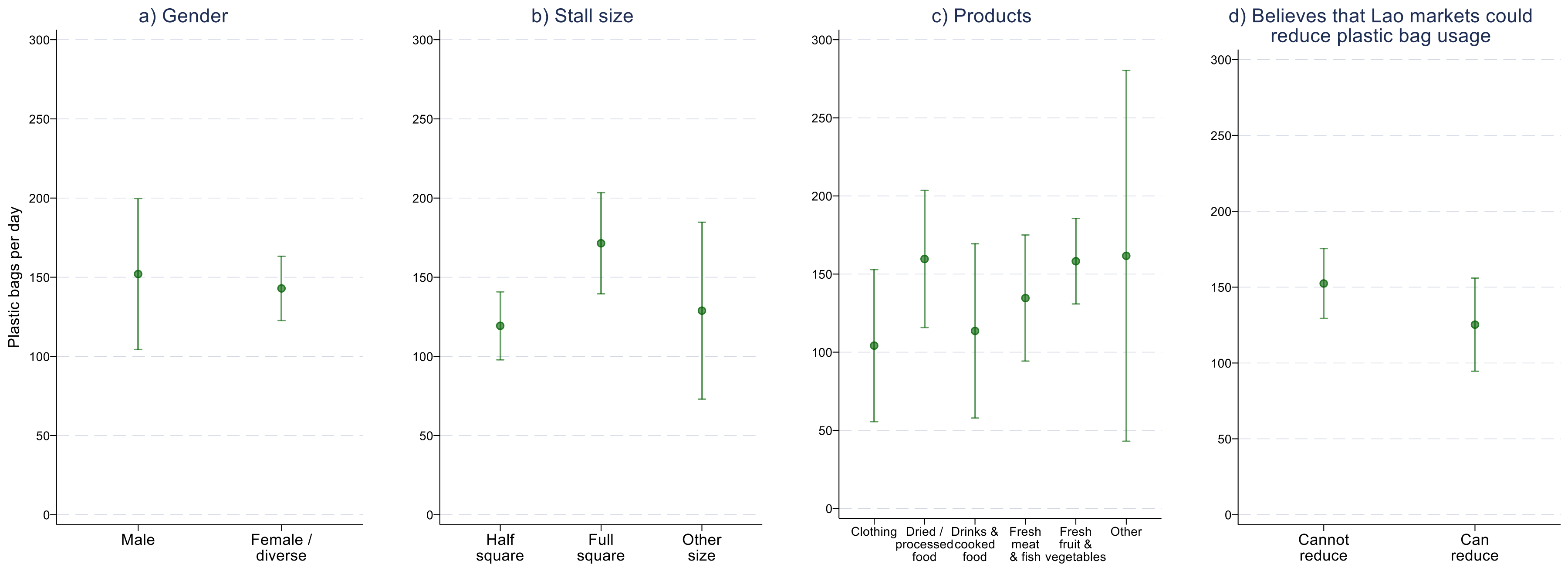

Notes. Bivariate relationships. Error bars indicate 95% confidence intervals. Stall size has a statistically significant relationship with daily plastic bag usage at the one percent level.

Predicted daily plastic bag use by vendors' stall size and main products

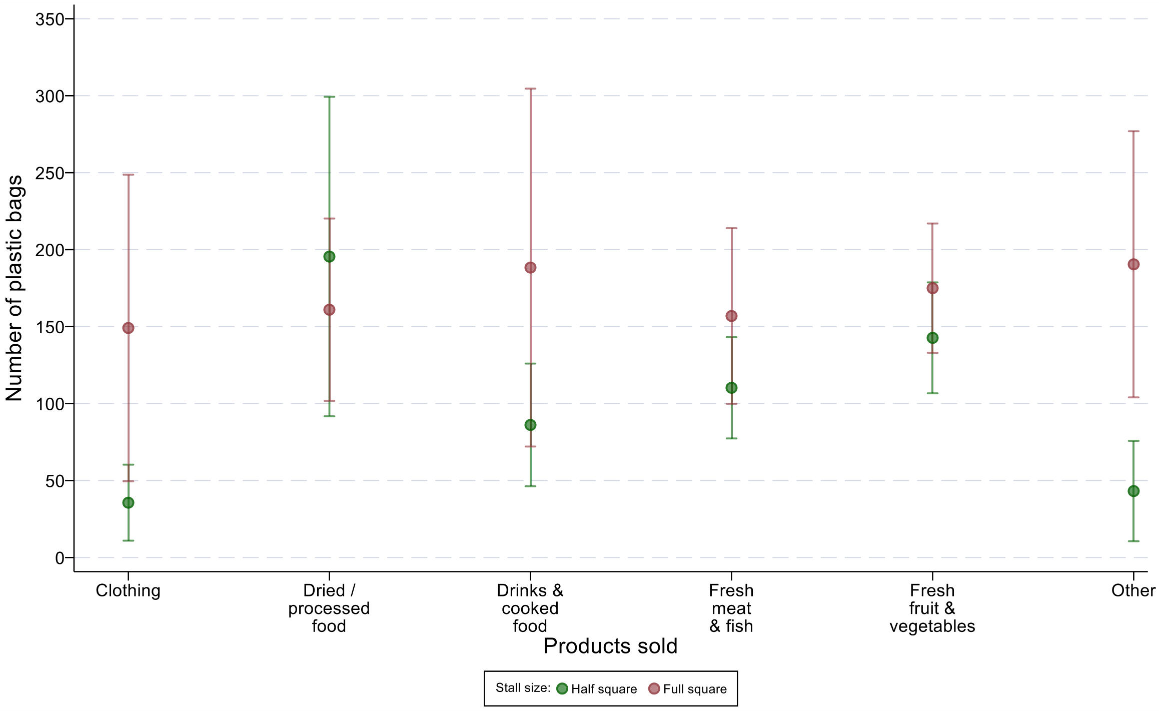

Notes. Multivariate relationships representing interaction between main product and stall size in negative binomial regression model controlling for age and gender of vendor. Error bars indicate 95% confidence intervals.

Statistical note:
Based on the qualitative observations, we specified a simple exploratory model with the number of daily plastic bags used per day by a stall as dependent variable, and, as independent variables, the type of products sold by the stall (categorical variable of clothing, dried or processed food, drinks and cooked food, fresh meat or fish, fresh vegetables and fruits, and other products), the size of the stall (binary variable of half or full square, omitting "other" kinds of sizes such as itinerant vendors), the age of the stall operator (quantitative variable in years), and the gender (binary variable of male or non-male) of the stall operator. We also considered that plastic bag usage varied differently across stall size and product types, as a result of which we included an interaction term to explore this relationship. Owing to the overdispersed count data of plastic bag usage (mean of 144.6 compared to a variance of 18,242.1), we choose a negative binomial regression model to estimate the relationship between the dependent and independent variables. The interaction model with 183 observations was statistically significant (p=0.002) and its likelihood ratio test indicated that α was statistically significantly different from zero at p<0.001, which supported the choice of a negative binomial over a Poisson regression model.[1,2] In addition, compared to a model without interaction term, the model with interaction term had a higher Pseudo R^2 and a lower Akaike Information Criterion (McFadden's Pseudo R^2: 0.015 vs. 0.009; Akaike Information Criterion: 2172.9 vs. 2177.2), which indicated that the interaction improved model goodness-of-fit.[3] The detailed model results are presented in the table below. To reduce complexity, and given that the model coefficients expressed in the log of expected counts are difficult to interpret on their own, we predicted the daily plastic bag use by vendors' stall size and their main products based on the regression results (controlling for vendor age and gender). The results are presented in the graph above. Please also note that the model results should be understood as indicative associations within the single-market case study site based on self-reported data; they can neither be understood as definitive empirical facts beyond the specific market nor as causal links.

Binomial regression model results of the relationship between daily plastic bag use and vendor attributes.

	Variable	No Interaction term	Interaction
Main products sold (ref: Clothing)	Dried or processed food	0.678** (0.303)	1.702*** (0.451)
	Drinks and cooked food	0.374 (0.328)	0.882** (0.430)
	Fresh meat or fish	0.445 (0.281)	1.129*** (0.384)
	Fresh vegetables and fruits	0.620** (0.273)	1.387*** (0.378)
	Other	0.422 (0.325)	0.192 (0.521)
Stall size: full square (ref: half square / small)		0.368*** (0.121)	1.431*** (0.486)
Age in years		-0.009* (0.005)	-0.008 (0.005)
Gender: non-male (ref: male)		-0.080 (0.155)	-0.137 (0.153)
Interaction term product x stall size (refs: Clothing, half square)	Dried or processed food		-1.625*** (0.589)
	Drinks and cooked food		-0.649 (0.622)
	Fresh meat or fish		-1.078** (0.543)
	Fresh vegetables and fruits		-1.227** (0.517)
	Other		0.053 (0.661)
Constant		4.700*** (0.337)	4.015*** (0.398)
ln(α)		-0.496*** (0.097)	-0.563*** (0.098)
Number of observations		183	183
Pseudo R^2		0.009	0.015
Model test (*p* value)		0.016	0.002
Akaike Information Criterion		2177.2	2172.9

Notes. Coefficients reported. Standard errors in parentheses
* $p < 0.10$, ** $p < 0.05$, *** $p < 0.01$

References

1. Hilbe, JM. (2011). *Negative binomial regression* (2nd ed.). Cambridge: Cambridge University Press.
2. Cameron, AC, & Trivedi, PK. (1998). *Regression analysis of count data*. Cambridge: Cambridge University Press.
3. Akaike, H. (1974). A new look at the statistical model identification. *IEEE Transactions on Automatic Control, 19*(6), 716-723.
